# Abh, AbrB3, and Spo0A play distinct regulatory roles during polymyxin synthesis in *Paenibacillus polymyxa* SC2

**DOI:** 10.1128/spectrum.02293-23

**Published:** 2023-12-06

**Authors:** Yanru Cui, Dongying Zhao, Kai Liu, Xiangui Mei, Shanshan Sun, Binghai Du, Yanqin Ding

**Affiliations:** 1 College of Life Sciences, Shandong Engineering Research Center of Plant-Microbia Restoration for Saline-Alkali Land, State Key Laboratory of Crop Biology, National Engineering Research Center for Efficient Utilization of Soil and Fertilizer Resources, Shandong Agricultural University, Tai'an, China; 2 State Key Laboratory of Crop Biology, College of Agronomy, Shandong Agricultural University, Tai'an, China; USDA-ARS-NPRL, Dawson, Georgia, USA

**Keywords:** polymyxin, *Paenibacillus polymyxa*, Abh, AbrB3, Spo0A

## Abstract

Polymyxins exhibit antibacterial activity against various gram-negative bacteria. However, the regulatory mechanisms of polymyxin synthesis remain unclear. Here, Abh, AbrB3, and Spo0A of *Paenibacillus polymyxa* SC2 were found to bind to P*_pmx_* via DNA pull-down and electromagnetic mobility shift assays. Oxford Cup and liquid chromatography-mass spectrometry assays showed that antibacterial activity and polymyxin production increased in the Δ*abh* strain. Overexpression of *abh* and *abrB3* significantly decreased the antibacterial activity and polymyxin production. The transcription of *pmxA* determined via quantitative reverse transcription-PCR supported these results. Moreover, the mutation and complementation of *spo0A* revealed that Spo0A was an activator of polymyxin synthesis. Spo0A could bind to the *abh* promoter to activate *abh* expression. Spo0A could bind to the *abrB3* promoter to inhibit *abrB3* transcription and mitigate the inhibitory effect of AbrB3 on *abh* transcription.

**IMPORTANCE:**

Polymyxins are considered the last line of defense against multidrug-resistant bacteria. The regulatory mechanism of polymyxin synthesis is poorly studied in *Paenibacillus polymyxa*. In this study, we found that Abh and AbrB3 negatively regulated, whereas Spo0A positively regulated polymyxin synthesis in *P. polymyxa* SC2. In addition, a regulatory relationship between Abh, AbrB3, and Spo0A was revealed, which regulate polymyxin synthesis via multiple regulatory mechanisms in *P. polymyxa*.

## INTRODUCTION

*Paenibacillus polymyxa* is a plant growth promoting rhizobacterium well-known for its growth-promoting and biocontrol effects in agriculture (1 – 3). Its biocontrol effects against phytopathogenic bacteria are mainly attributed to polymyxin secretion (4, 5). Polymyxin is a cationic cyclic peptide that was first identified in 1947 (6). Polymyxin P is the main metabolite produced by *P. polymyxa* M1, which inhibits the growth of phytopathogenic *Erwinia* spp. (7). Polymyxins B_1_ and E_2_ of *P. polymyxa* Y-1 exert good biocontrol effects against rice bacterial blight and bacterial leaf streak (8). In addition, polymyxins exhibit therapeutic effects against multidrug-resistant gram-negative bacteria and are considered the last line of defense against multidrug-resistant bacteria (9 – 11).

The polymyxin synthase gene cluster, which is approximately 40 kb in size, consists of three synthase (*pmxA*, *pmxB*, and *pmxE*) and two ABC transporters (*pmxC* and *pmxD*) genes. Mutations in *pmxE* of *P. polymyxa* PKB1 lead to a loss of polymyxin production (7, 12, 13). Although polymyxin synthesis has been widely studied, its regulatory mechanisms remain unclear. Most studies on the regulation of polymyxin synthesis have focused on *Bacillus subtilis* rather than *P. polymyxa* because of the difficulty of performing molecular experiments on *P. polymyxa* (14, 15). AbrB has been found to directly inhibit polymyxin synthesis, and Spo0A is required for polymyxin synthesis in *B. subtilis* (15).

*P. polymyxa* SC2 was previously screened from the rhizosphere of pepper plants cultivated in Guizhou Province, China, and has been reported to exhibit growth promotion and biological control activities (16, 17). Whole-genome sequencing of SC2, which contains the polymyxin synthase gene cluster (*pmxA-E*) via anti-SMASH analysis, was first completed in 2010 (17, 18). *P. polymyxa* is susceptible to morphological changes and functional degradation during culturing (19, 20). We previously reported that SC2 can be differentiated into two morphologically distinct strains, SC2-M1 and SC2-M2, both of which lose the ability to form spores (19). As the wild-type strain SC2 cannot be easily manipulated and is susceptible to developing spontaneous mutations, the strain SC2-M1 was previously used for molecular manipulation to study the regulatory mechanisms of polymyxin synthesis in *P. polymyxa*. In our previous study, we successfully knocked out *msmR1* in SC2-M1 and demonstrated that MsmR1 (a global transcriptional regulator) indirectly affects polymyxin synthesis (21).

In this study, we screened and identified transcription factors related to polymyxin biosynthesis and revealed their regulatory mechanisms. Abh and AbrB3 directly inhibited polymyxin synthesis, whereas Spo0A directly activated polymyxin synthesis. In addition, electromagnetic mobility shift assay (EMSA) and quantitative reverse transcription-PCR (qRT-PCR) showed that Spo0A directly inhibited the expression of *abrB3* and activated the expression of *abh*, whereas AbrB3 and Abh inhibited the expression of each other by directly binding to their promoters. Collectively, these findings provide new insights into the regulation of polymyxin synthesis.

## RESULTS

### Transcriptional activity of promoter P*_pmx_*

**Fig 1:**
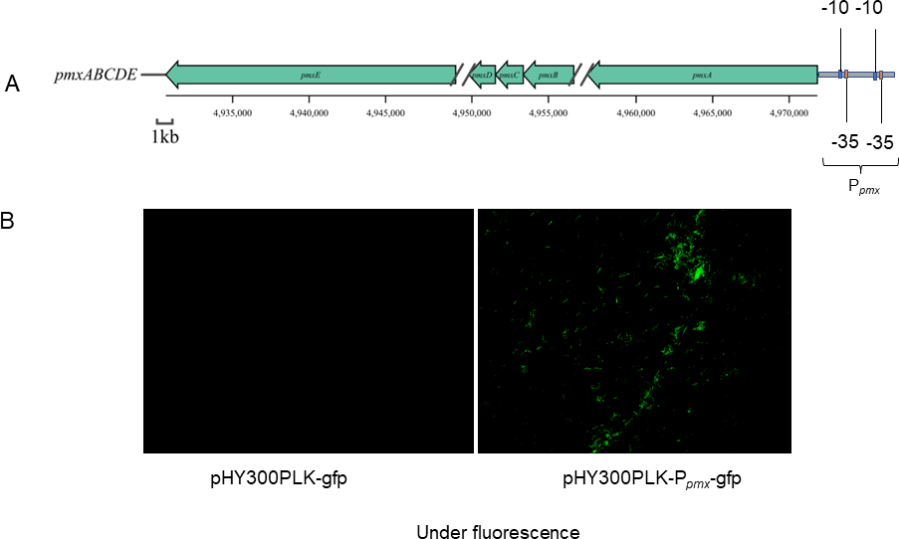
The transcriptional activity of the promoter P*_pmx_*. (**A**) The schematic diagram of polymyxin synthetase gene cluster in *P. polymyxa* SC2. (**B**) Fluorescence intensities of SC2-M1 strain carrying the pHY300PLK-gfp and pHY300PLK-P*_pmx_*-gfp fusions, respectively (at 1,000× magnification).

*P. polymyxa* SC2 contained five open reading frames consisting of a polymyxin synthetase gene cluster with a size of 41.2 kb (Fig. 1A). We performed promoter prediction for the upstream region of *pmxA* using Softberry and identified two −35 (TTTAAG; TTGAGC) and −10 regions (GCGTAGAAC; TCGTCACAT) of P*_pmx_* (Fig. S1A). To monitor the transcriptional activity of P*_pmx_*, a reporter fusion vector of P*_pmx_* and GFP was constructed and introduced into SC2-M1 cells. Fluorescence intensity detected using a fluorescence microscope indicated that this region has transcriptional activity (Fig. 1B).

### Candidate P*_pmx_*-binding proteins

**Fig 2:**
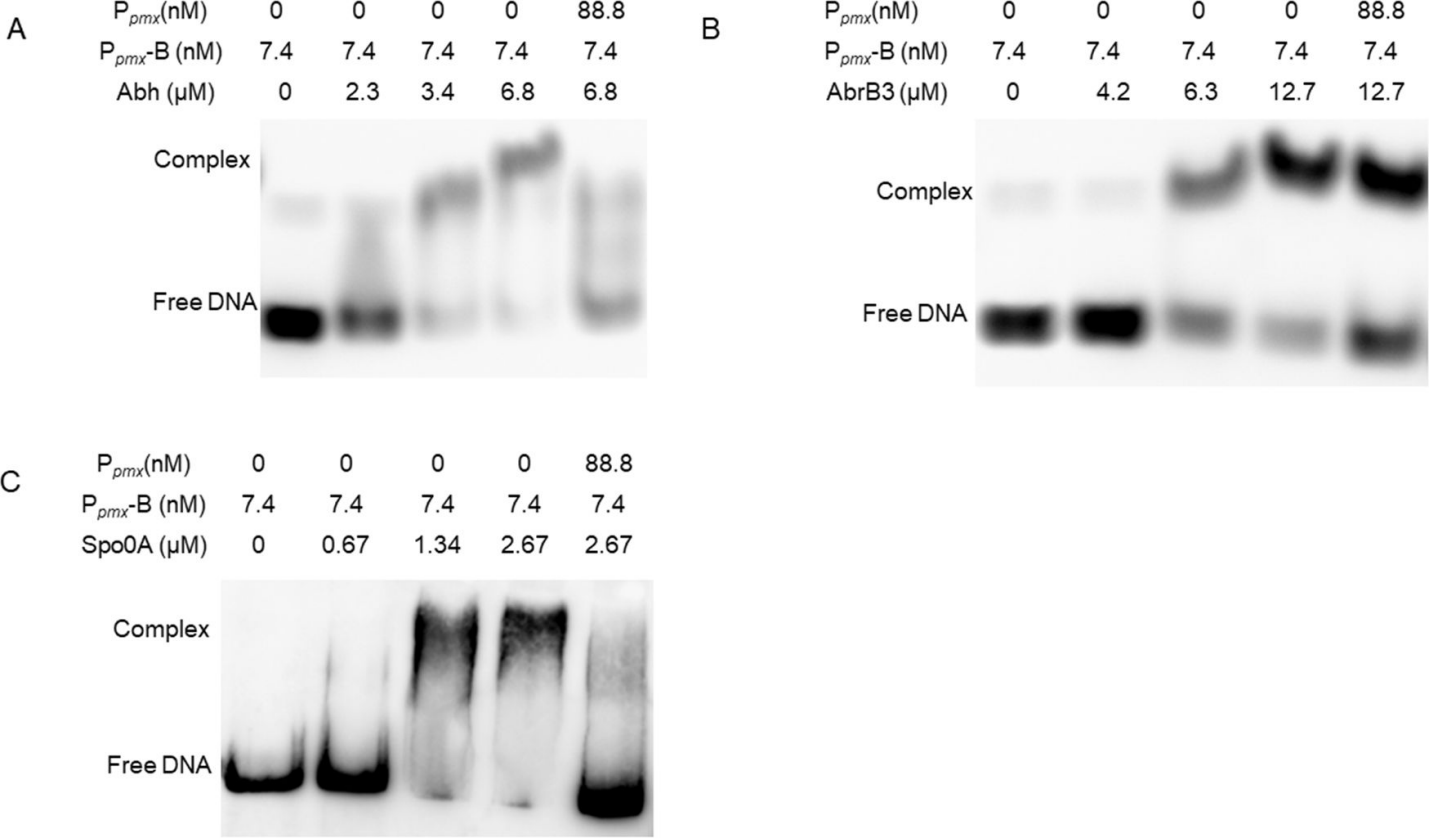
Identification of proteins that bind to P*_pmx_*. (**A**) The increasing concentrations of purified Abh (0, 2.3, 3.4, and 6.8 µM) binding to P*_pmx_* (7.4 nM) analyzed by EMSA. (**B**) The increasing concentrations of purified AbrB3 (0, 4.2, 6.3, and 12.7 µM) binding to P*_pmx_* (7.4 nM) analyzed by EMSA. (**C**) The increasing concentrations of purified Spo0A (0, 0.67, 1.34, and 2.67 µM) binding to P*_pmx_* (7.4 nM) analyzed by EMSA.

In this study, we aimed to identify key transcription factors involved in the activation or inhibition of polymyxin synthesis by performing DNA pull-down assay. Antibacterial activity was detected when the strain entered the exponential phase at 5 h, followed by a gradual increase in activity (Fig. S2). Therefore, we collected SC2 cells cultured for 5 and 10 h to extract the total protein, which was then used in the DNA pull-down assay. The primer pair pmxABF/R was used to amplify biotin-labeled DNA fragments. Both biotin-labeled DNA fragments and nuclease-free water (negative control) were incubated with beads, as described in the Methods section. Candidate P*_pmx_*-binding proteins were analyzed using LC-MS/MS. LC-MS/MS analysis showed that 10 candidate P*_pmx_*-binding proteins were specifically detected in the BL_5h_ group and 25 candidate P*_pmx_*-binding proteins were specifically detected in the BL_10h_ group compared to those in NF group (Table S2). ResD3, YlzA, and FruR1 were present in groups BL_5h_ and BL_10h_, and 32 candidate transcription factors were identified. Both Abh (detected in the BL_10h_ group) and AbrB3 (detected in the BL_5h_ group) were found to contain an AbrB domain similar to the transition state regulatory protein AbrB of *B. subtilis* (22). Previous studies have shown that AbrB can bind to the promoter of *pmx* cluster in *P. polymyxa* E681 to negatively regulate polymyxin synthesis (15). In addition, as shown in Fig. S1A, P*_pmx_* contains three 0A-like boxes (TGCCGAA; CGTAGAA; TGGCGAA) and one 0A-box: 5′-TTCGACA-3′ (5′-TGTCGAA-3′, complementary sequence) (23) and Spo0A was detected in both BL_10h_ and NF_10h_ groups. Therefore, we focused on *abh*, *abrB3,* and *spo0A* in this study. The proteins His6-Abh, His6-AbrB3, and His6-Spo0A were purified (Fig. S3) and analyzed via EMSA, which showed that Abh, AbrB3, and Spo0A were directly bound to P*_pmx_* (Fig. 2).

### Abh inhibited polymyxin synthesis

**Fig 3:**
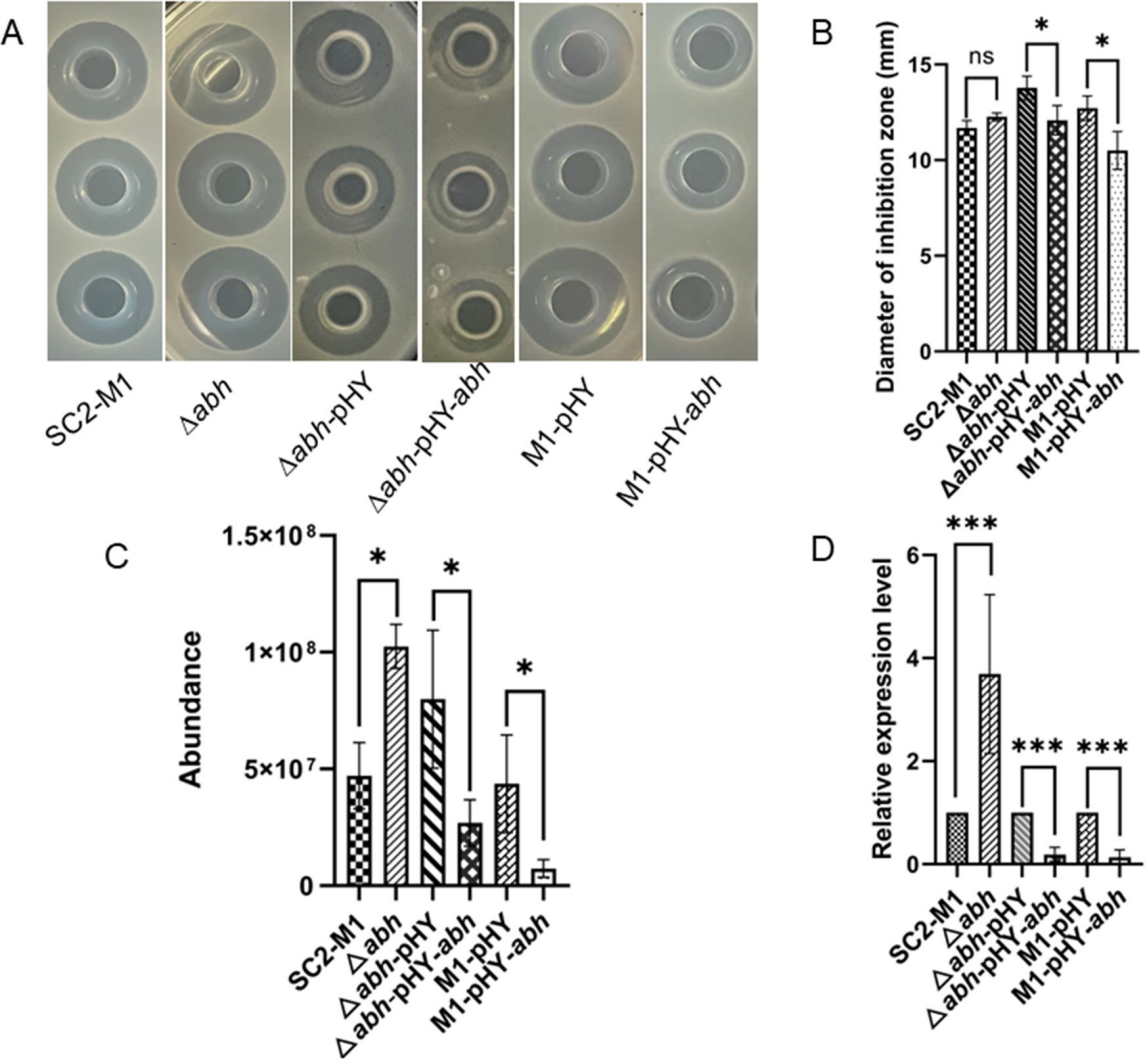
Abh inhibits polymyxin synthesis. The strains SC2-M1, Δ*abh*, Δ*abh*-pHY, Δ*abh*-pHY-*abh*, M1-pHY, M1-pHY-*abh* were cultured in fermentation medium for 24 h, respectively. Three biological repetitions for each group. (**A**) Antibacterial activities of strains SC2-M1, Δ*abh*, Δ*abh*-pHY, Δ*abh*-pHY-*abh*, and M1-pHY, and M1-pHY-*abh* against *Escherichia coli* DH5α detected by using Oxford Cup method. (**B**) The diameter of inhibition zone of strains SC2-M1, Δ*abh*, Δ*abh*-pHY, Δ*abh*-pHY-*abh*, M1-pHY, and M1-pHY-*abh* against *E. coli* DH5α. (**C**) The quantitative analysis of polymyxin in strains SC2-M1, Δ*abh*, Δ*abh* -pHY, Δ*abh* -pHY-*abh*, M1-pHY, and M1-pHY-*abh* by LC/MS. (**D**) The relative expression levels of *pmxA* in strains SC2-M1, Δ*abh*, Δ*abh* -pHY, Δ*abh* -pHY-*abh*, M1-pHY, and M1-pHY-*abh* determined by qRT-PCR. *, *P* < 0.05; **, *P* < 0.01; ***, *P* < 0.001.

To elucidate the mechanism by which Abh regulates polymyxin synthesis, we constructed a deletion, complementation, and overexpression strain of *abh*. Our data showed that the mutation in *abh* led to a minor increase in antimicrobial activity, whereas it significantly increased polymyxin content (Fig. 3A through C). qRT-PCR analyses revealed that the relative transcription level of *pmxA* was significantly increased in strain Δ*abh* (Fig. 3D). These changes were reversed by introducing pHY-*abh* into strain Δ*abh* (Fig. 3). However, when *abh* was overexpressed in strain SC2-M1, the antibacterial activity, polymyxin content, and the relative transcription level of *pmxA* were significantly decreased (Fig. 3A through D). Therefore, Abh effects polymyxin production by inhibiting transcription of the polymyxin synthetase gene cluster.

### AbrB3 inhibited polymyxin synthesis

**Fig 4:**
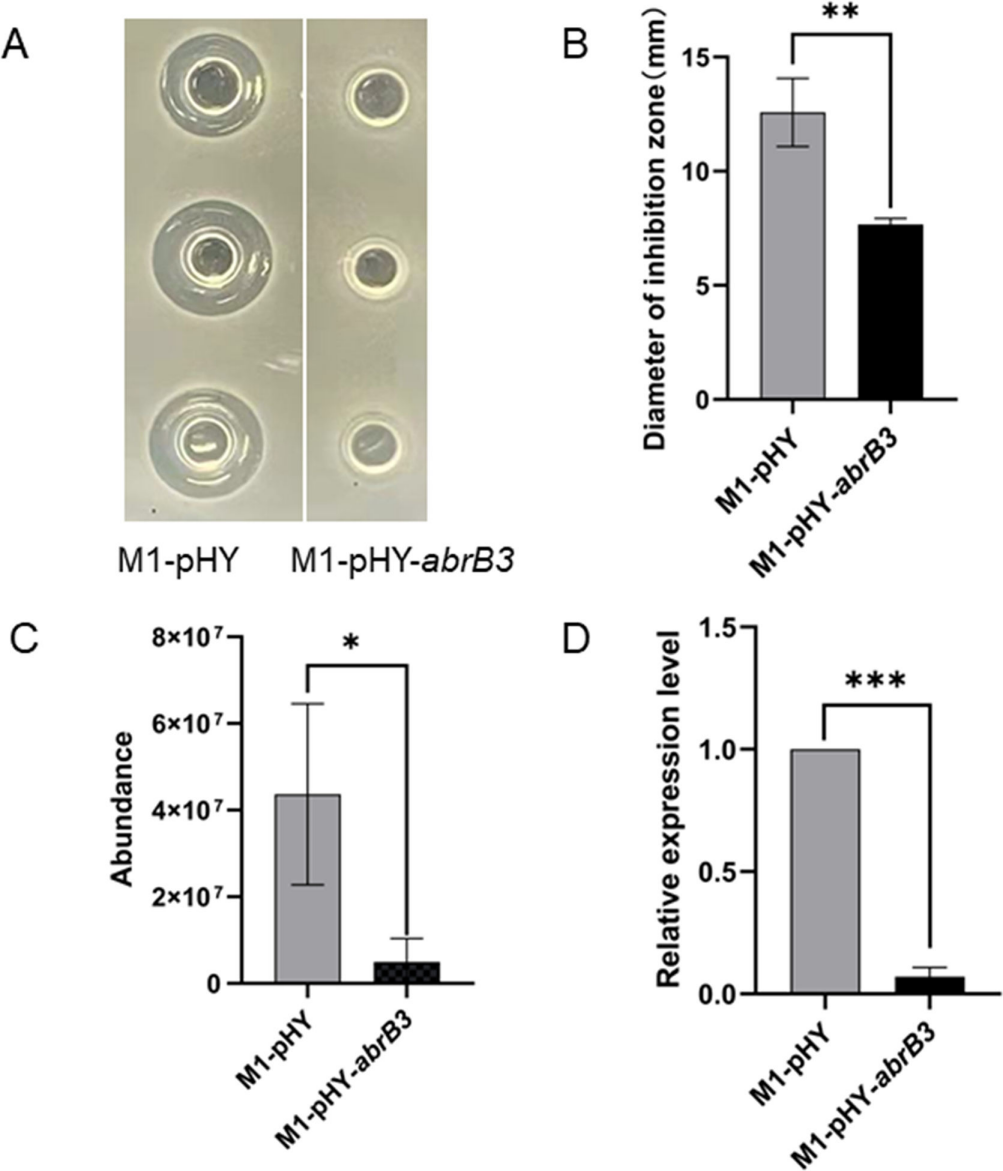
AbrB3 inhibits polymyxin synthesis. The strains M1-pHY, M1-pHY-*abrB3* were cultured in fermentation medium for 24 h, respectively. Three biological repetitions for each group. (**A**) Antibacterial activities of M1-pHY and M1-pHY-*abrB3* on *E. coli* DH5α detected by using Oxford Cup method. (**B**) The diameter of inhibition zone of M1-pHY and M1-pHY-*abrB3* against *E. coli* DH5α. (**C**) The quantitative analysis of polymyxin in strains M1-pHY and M1-pHY-*abrB3* detected by LC/MS. (**D**) The relative expression levels of *pmxA* in strains M1-pHY and M1-pHY-*abrB3* determined by qRT-PCR analysis. *, *P* < 0.05; **, *P* < 0.01; ***, *P* < 0.001.

To determine the relationship between AbrB3 and polymyxin synthesis *in vivo*, we constructed the overexpression strain M1-pHY-*abrB3*. The *abrB3* overexpression strain exhibited relatively weaker antibacterial activity against *E. coli* DH5α (Fig. 4A and B). Moreover, polymyxin production and the relative transcription level of *pmxA* were significantly decreased in the M1-pHY-*abrB3* strain (Fig. 4C and D), indicating that AbrB3 inhibited polymyxin synthesis.

### Spo0A positively regulated polymyxin synthesis

**Fig 5:**
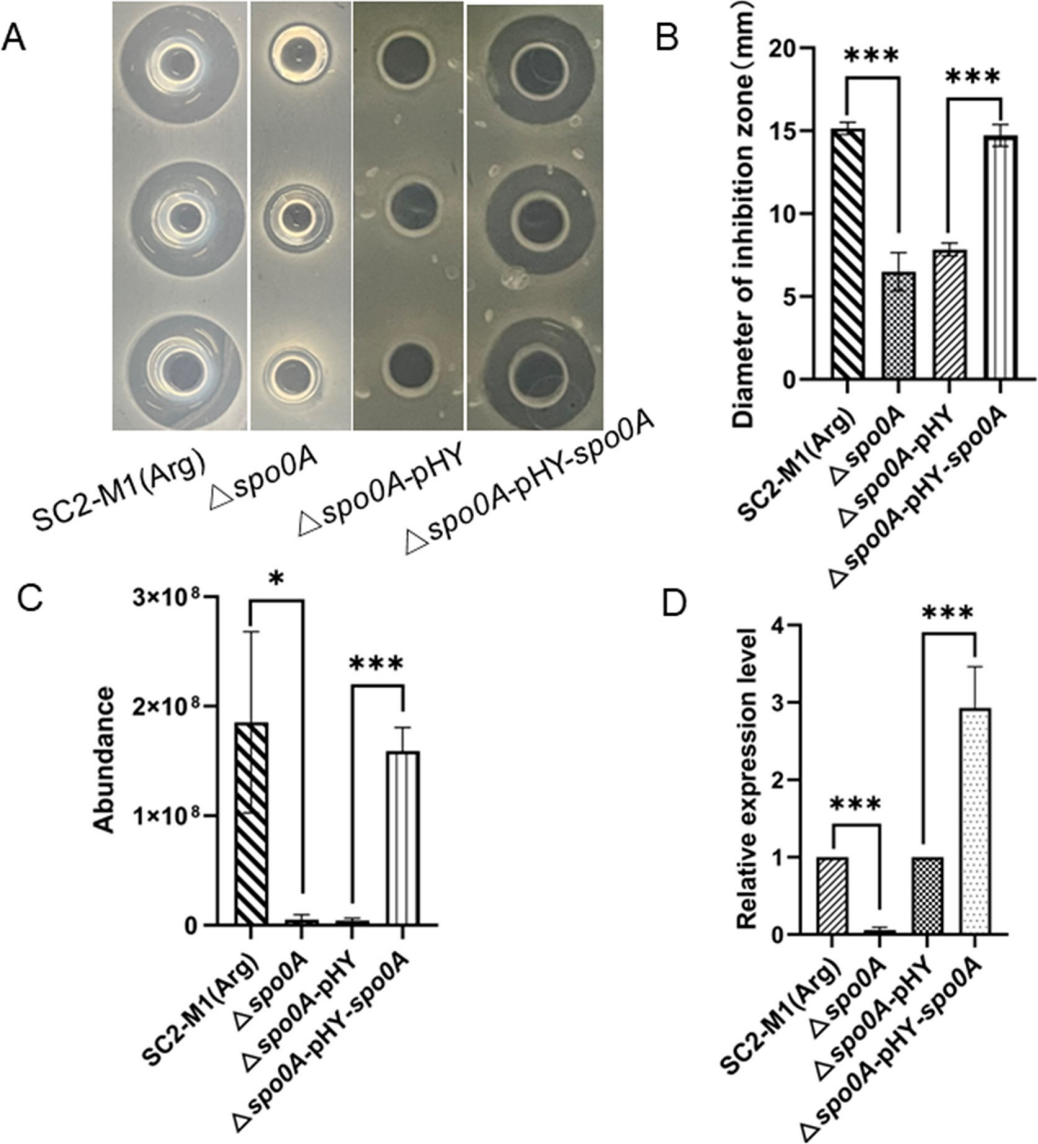
Spo0A positively regulates polymyxin biosynthesis. The strains SC2-M1(Arg), Δ*spo0A*, Δ*spo0A*-pHY, and Δ*spo0A*-pHY-*spo0A* were cultured in fermentation medium for 24 h, respectively. Three biological repetitions for each group. (**A**) Antibacterial activities of SC2-M1(Arg), Δ*spo0A*, Δ*spo0A*-pHY, and Δ*spo0A*-pHY-*spo0A* strains on *E. coli* DH5α detected by using Oxford Cup method. (**B**) The diameter of inhibition zone of SC2-M1(Arg), Δ*spo0A*, Δ*spo0A*-pHY, and Δ*spo0A*-pHY-*spo0A* strains against *E. coli* DH5α. (**C**) The quantitative analysis of polymyxin in strains SC2-M1(Arg), Δ*spo0A*, Δ*spo0A*-pHY, and Δ*spo0A*-pHY-*spo0A* strains detected by LC/MS. (**D**) The relative expression levels of *pmxA* in strains SC2-M1(Arg), Δ*spo0A*, Δ*spo0A*-pHY, and Δ*spo0A* -pHY-*spo0A* detected by qRT-PCR analysis. *, *P* < 0.05; **, *P* < 0.01; ***, *P* < 0.001.

To determine the mechanism via which Spo0A affects polymyxin synthesis, we constructed *spo0A* knockout and complementation strains. Next, we determined the polymyxin content of the *spo0A* mutant and its antimicrobial activity against *E. coli* DH5α. Compared with the SC2-M1(Arg) strain (reversion of Spo0A in which the R211H substitution occurred in the HTH region), the antimicrobial activity almost disappeared and the polymyxin content distinctly decreased in the Δ*spo0A* strain (Fig. 5A through C). Furthermore, the relative transcription level of *pmxA* in strain Δ*spo0A* was significantly lower than that in the SC2-M1(Arg) strain (Fig. 5D). Both the polymyxin production and the relative transcription level of *pmxA* were restored by pHY-*spo0A* into Δ*spo0A* strain (Fig. 5). Therefore, Spo0A positively regulated polymyxin synthesis.

### Regulatory relationship of Abh, AbrB3, and Spo0A

**Fig 6:**
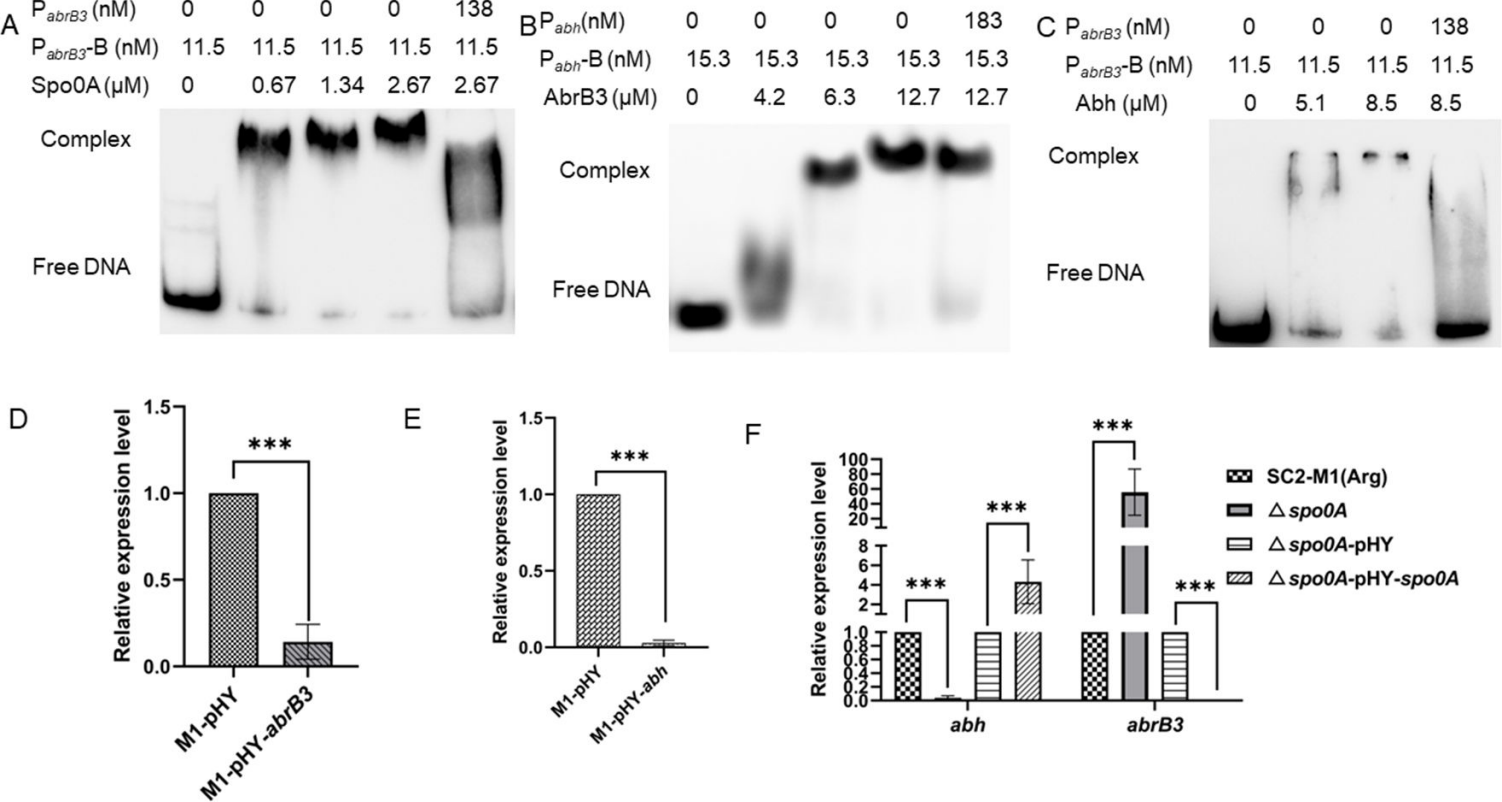
The regulatory relationship of Abh, AbrB3, and Spo0A. The strains M1-pHY, M1-pHY-*abrB3*, Δ*spo0A*, SC2-M1(Arg), Δ*spo0A*-pHY, and Δ*spo0A*-pHY-*spo0A* were cultured in fermentation medium for 24 h, respectively. Three biological repetitions for each group. (**A**) The increasing concentrations of Spo0A (0, 0.67, 1.34, and 2.67 µM) binding to P*_abrB3_* (11.5 nM) analyzed by EMSA. (**B**) The increasing concentrations of AbrB3 (0, 4.2, 6.3, and 12.7 µM) binding to P*_abh_* (15.3 nM) analyzed by EMSA. (**C**) The increasing concentrations of Abh (0, 5.1, and 8.5 µM) binding to P*_abrB3_* (11.5 nM) analyzed by EMSA. (**D**) The relative expression level of *abh* in strains M1-pHY-*abrB3* and M1-pHY detected by qRT-PCR. (**E**) The relative expression level of *abrB3* in strains M1-pHY-*abh* and M1-pHY detected by qRT-PCR. (**F**) The relative expression levels of *abh* and *abrB3* in strains SC2-M1(Arg), Δ*spo0A*, Δ*spo0A-*pHY, and Δ*spo0A-*pHY-*spo0A* detected by qRT-PCR analysis. *, *P* < 0.05; **, *P* < 0.01; ***, *P* < 0.001.

Spo0A inhibits the transcription of *abrB,* while AbrB inhibits the expression of *abh* in *B. subtilis* (24). However, the mechanisms underlying the regulation of expression between these three genes in *P. polymyxa* remain unclear. First, we performed promoter prediction for the upstream region of *abh*, *abrB3,* and *spo0A* using Softberry (Fig. S1B through D). Subsequently, EMSA was performed, which showed that Spo0A was bound to P*_abrB3_*, P*_abh_*, and P*_spo0A_*, AbrB3 was bound to P*_abh_* and P*_abrB3_*, and Abh was bound to P*_abrB3_* (Fig. 6A through C; Fig. S4). In addition, qRT-PCR revealed that the relative transcription level of *abh* was significantly decreased in the overexpression strain M1-pHY-*abrB3* (Fig. 6D), and the relative transcription level of *abrB3* was significantly decreased in the overexpression strain M1-pHY-*abh* (Fig. 6E). In addition, the relative expression level of *abrB3* was significantly increased and that of *abh* was significantly decreased in the Δ*spo0A* strain compared to those in SC2-M1(Arg) (Fig. 6F). In summary, Spo0A positively regulated *abh* expression by binding to the *abh* promoter and indirectly regulated *abh* expression by directly inhibiting the transcription of *abrB3*. Spo0A and AbrB3 were found to be self-regulated.

## DISCUSSION

**Fig 7:**
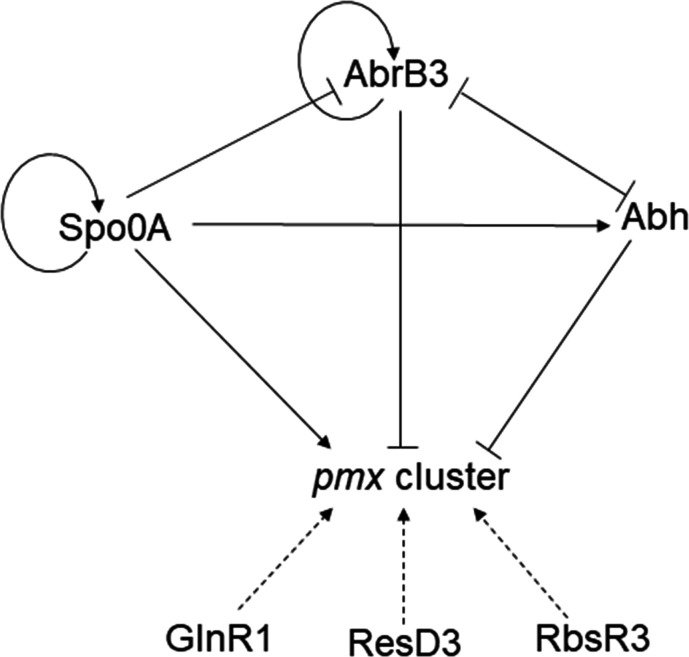
Proposed the regulatory model of Spo0A, AbrB3, and Abh in polymyxin synthesis. Abh and AbrB3 directly inhibited polymyxin production by binding to the *pmx* cluster promoter. Spo0A directly or indirectly promoted polymyxin production by binding to the promoter of *pmx* cluster and *abrB3.* AbrB3 and Abh inhibited each other by directly binding to their respective promoters. The regulatory effects of ResD3, RbsR3, and GlnR1 on polymyxin synthesis require further investigation. Arrows represent activation; bar-ended lines represent inhibition; curved arrows represent self-regulated; dotted arrows represent uncertain regulation.

Although the polymyxin synthase gene cluster has been identified in several strains, the mechanisms of underlying polymyxin synthesis regulation remain unclear (5, 7, 8, 12). In the present study, we identified multiple candidate transcription factors that could bind to the promoter region of the *pmx* cluster based on DNA pull-down assays (Table S2). Using the promoter of *yfr2* as a bait, the candidate transcription factor PMM1637 was screened using DNA pull-down assay (25). Our results indicated that this method is effective in identifying transcription factors. Based on our data, the regulatory networks of Abh, AbrB3, and Spo0A were proposed (Fig. 7). Abh and AbrB3 were directly inhibited, whereas Spo0A directly and indirectly activated the expression of the *pmx* cluster in *P. polymyxa* SC2. AbrB3 and Abh inhibit each other by directly binding to their respective promoters. Spo0A and AbrB3 were found to be self-regulated. However, the regulatory effects of ResD3, RbsR3, and GlnR1 on polymyxin synthesis require further investigation.

In *B. subtilis*, AbrB is a transition-state regulator, and the monomer comprises an N-terminal domain (DNA-binding) and a C-terminal domain (multimerization). On the other hand, Abh is a paralogous protein of AbrB showing high similarity with regard to the N-terminal domain (26, 27). Abh and AbrB mostly function as repressor that regulate gene expression, but may perform contradictory functions in certain cases (24, 28). Abh and AbrB regulate the expression of many antibiotic synthesis genes, including *sdpABC*, *skfABCDEFGH*, *sboA*, and *sunA* (24, 29, 30). Although the regulatory functions of Abh and AbrB have been extensively studied in *B. subtilis*, their functions in *P. polymyxa* polymyxin synthesis remain unclear. In this study, both Abh and AbrB3 were found to possess an AbrB domain similar to AbrB in the N-terminal domain of *B. subtilis,* and both could bind to P*_pmx_* based on EMSA (Fig. 2A and B) (22). Moreover, the *abh* knockout increased polymyxin production and *pmxA* expression compared to those in SC2-M1 cells, which were restored by introducing pHY-*abh* into Δ*abh* (Fig. 3). In addition, antibacterial activity, polymyxin production, and *pmxA* expression decreased significantly in *abh* and *abrB3* overexpression strains (Fig. 3 and 4). We could not study the regulatory function of AbrB3 *in vivo* further owing to the absence of an *abrB3* mutant. However, the *abrB* mutant has been reported to show higher production of polymyxin when heterologously expressing the polymyxin synthase gene cluster in *B. subtilis* (15). In summary, Abh and AbrB3 are negative regulators that directly inhibit polymyxin synthesis in *P. polymyxa* SC2.

P*_pmx_* contained three 0A-like boxes (TGCCGAA; CGTAGAA; TGGCGAA) and one 0A-box: 5′-TTCGACA-3′ (5′-TGTCGAA-3′, complementary sequence) (Fig. S1A). However, it is unclear whether Spo0A directly regulates the expression of this gene cluster. In this study, EMSA revealed that Spo0A was bound to P*_pmx_*, indicating that Spo0A directly regulates polymyxin synthesis (Fig. 2C). Previous studies have shown that polymyxin production is Spo0A-dependent because the *spo0A* mutant strain cannot produce polymyxin in *P. polymyxa* E681, and Spo0A indirectly and positively regulates the polymyxin synthesis in *B. subtilis* (15). Here, the antibacterial activity and *pmxA* expression were significantly decreased in the *spo0A* mutant (Fig. 5A and D). LC/MS results showed that the *spo0A* mutant did not completely lose its ability to produce polymyxin (Fig. 5C). Therefore, Spo0A positively and directly regulates polymyxin synthesis in *P. polymyxa* SC2. In this study, compared to the knockout strain Δ*spo0A*, the SC2-M1 strain showed efficient polymyxin production, although polymyxin synthesis in strain SC2-M1 was affected to a certain extent. The relative transcription level of *abh* was significantly decreased (log2FC = −4.61) in SC2-M1 compared to that in SC2 based on transcriptomic analysis (19). We suggest that Spo0A (R211H substitution occurring in the HTH region of Spo0A) in SC2-M1 positively regulates *pmx* expression and suppresses the inhibitory effect of Abh on *pmx* expression. A mutation at position 257 of Spo0A results in loss of sporulation in the strain; Spo0A (A257V) can still inhibit the transcription of *abrB* (31). In the present study, whether or not Spo0A (R211H) can combine with P*_pmx,_* P_*abh*_ and P*_abrB3_* needs further investigation. In addition, we demonstrated that other candidate binding proteins (ResD3, RbsR3, GlnR1) (data not shown) bind to P*_pmx_* except Abh, AbrB3, and Spo0A, indicating that these proteins may also regulate the expression of the *pmx* cluster.

In *B. subtilis*, Spo0A directly inhibits *abrB* expression, whereas AbrB inhibits *abh* expression (24, 32). In *P. polymyxa* WLY78, Spo0A directly inhibits *abrB* expression (33). Here, our EMSA and qRT-PCR results indicated that AbrB3 and Abh inhibited each other by directly binding to their respective promoters; Spo0A could bind to the *abh* promoter to activate *abh* expression. Spo0A could bind to the *abrB3* promoter to inhibit *abrB3* transcription and mitigate the inhibitory effect of AbrB3 on *abh* transcription (Fig. 6; Fig. S4). AbrB is a transition phase regulator that mainly plays a regulatory role in the exponential growth of strains (28, 34). AbrB levels decrease as the strain enters the stationary phase from the growth phase, whereas Spo0A levels increase after the exponential phase (35). In the present study, only AbrB3 was found in the BL_5h_ group, whereas Abh, AbrB3, and Spo0A were found in the BL_10h_ group; AbrB3 and Spo0A were also found in the NF_10h_ group. Based on these findings, we hypothesize that AbrB3 represents the main inhibitor of polymyxin synthesis at the early stage of strain growth, and that Spo0A combined with Abh dynamically regulates *pmxA* expression at the later growth stage of the strain. This hypothesis requires further verification in future studies.

In the present study, Abh and AbrB3 directly inhibited the expression, whereas Spo0A both directly and indirectly activated the expression of the *pmx* cluster in *P. polymyxa* SC2. We revealed a regulatory relationship between Abh, AbrB3, and Spo0A, which regulate polymyxin synthesis via multiple regulatory mechanisms in *P. polymyxa*. By studying the regulatory mechanism of polymyxin synthesis, we can provide a theoretical foundation for the future metabolic engineering of strains. To achieve this, proteomics and transcriptomic analyses are required to study the regulatory mechanism. Genes that encode negative regulator proteins can be knocked out, and genes that encode positive regulator proteins can be overexpressed to construct high-yield strains.

## MATERIALS AND METHODS

### Strains and growth conditions

All strains, plasmids, and primers used in this study are listed in Table S1. SC2 was cultured in a medium (g/L) consisting of 20 g sucrose, 5 g (NH_4_)_2_SO_4_, 0.2 g NaCl, 3.68 g K_2_HPO_4_, 1.32 g KH_2_PO_4_, and 0.2 g MgSO_4_ for total protein extraction for DNA pull-down assay. Fermentation medium (g/L) for polymyxin synthesis consisted of 40 g sucrose, 8 g (NH_4_)_2_SO_4_, 0.2 g NaCl, 0.2 g KH_2_PO_4_, 0.2 g MgSO_4_, and 5 g CaCO_3_. Luria-Bertani (LB) medium (g/L) containing 10 g NaCl, 10 g tryptone, and 5 g yeast extract was used in other experiments. The following antibiotics were used in this study: 100 µg/mL of ampicillin, 50 µg/mL of kanamycin, 12.5 µg/mL erythromycin, 20 µg/mL tetracycline, and 6 µg/mL of chloramphenicol. Unless otherwise noted, all strains were cultured at 37°C.

### Promoter activity verification

The pmxAF/AR primer pair was used to amplify P*_pmx_* from the genomic DNA (gDNA) of SC2. After digestion of plasmid pHY300PLK-P*_gap_*-gfp-cm using *Xba*I and *Bam*HI, it was ligated with the amplified fragment at 16°C using T4 DNA ligase (Accurate Biotechnology, Hunan, Co., Ltd., Changsha, China) for 12 h and then transformed into *Escherichia coli* DH5α and SC2-M1 (19, 36). The strains carrying a GFP-fusion vector were cultured in LB solid medium containing 20 µg/mL tetracycline and cultured at 37°C for 24 h. Single colonies were selected to prepare bacterial suspensions, and fluorescence activity was observed under a fluorescence microscope at 1,000× magnification (Olympus BX53, Japan).

### DNA affinity chromatography-pulldown (DNA pull-down) assay

The DNA pull-down assay was performed as previously described (37). Briefly, the collected SC2 cells (cultured for 5 and 10 h) were washed twice with phosphate-buffered saline (PBS) (0.01 M PO_4_^3-^, 0.8% NaCl, 0.02% KCl, pH 7.2–7.4) and resuspended in BS/THES buffer for sonication (37), and the total protein content in the supernatant was determined after centrifugation. The fragment P*_pmx_* was amplified via PCR using a 5′-biotinlabed primer (pmxABF/pmxABR). Biotin-labeled DNA fragments (200 ng/µL) were combined with pretreated Dynabeads M-280 Streptavidin (Invitrogen, Carlsbad, CA, USA) at room temperature for 30 min in a rotor for an experimental group named BL. The aforementioned step was repeated. Biotin-labeled DNA fragments (200 ng/µL) were replaced with nuclease-free water, and similar incubation with beads was performed in the negative control group named NF. Biotin-labeled DNA fragment bead complex incubation with the aforementioned total protein (cultured for 5 and 10 h named BL_5h_ and BL_10h_, respectively) and beads (incubation with nuclease-free water) incubation with above total protein (cultured for 5 and 10 h named NF_5h_ and NF_10h_, respectively) were performed separately. The bait bound to the target protein was eluted using different concentrations of 100, 200, 300, 500, 750, 1,000 mM of NaCl solution. The protein eluent was digested with trypsin and analyzed via liquid chromatography-mass spectrometry (LC-MS/MS) on a Q Exactive (Thermo Fisher Scientific, San Jose, CA, USA) coupled with Easy-nLC 1000 (Thermo Fisher Scientific, San Jose, CA, USA). LC-MS/MS was performed by Shanghai Applied Protein Technology Co., Ltd. (Shanghai, China). LC-MS/MS data were analyzed using mascot 2.2 to obtain qualitative identification information of the target protein polypeptides.

### Expression and purification of transcription factors

Primer pairs abrB3-PF/PR, abh-PF/PR, and spo0A-PF/PR were used to amplify the target fragments from gDNA of SC2. After digesting the plasmid pET-28b(+) using *Sal*I and *Xho*I, it was ligated with the target fragment using the ClonExpress MultiS One Step Cloning Kit (Vazyme Biotech, China), transformed into *E. coli* DH5α, and subsequently transformed into *E. coli* BL21 (DE3). BL21-pET28b(+)-*abrB3*, BL21-pET28b(+)-*abh*, and BL21-pET28b(+)-*spo0A* were cultured and then inoculated into 60 mL LB medium (containing 50 µg/mL kanamycin) at a 1:50 dilution. When the optical density at 600 nm (OD_600_) reached 0.6, isopropyl-β-D-thiogalactopyranoside was added (final concentration: 0.6 mM) to culture at 16°C for 18 h. The cells were collected, washed twice with 1× PBS, and resuspended for sonication. Protein purification was performed using His-tag Purification Resin (Beyotime, China) according to the manufacturer’s instructions. Purified proteins were analyzed by sodium dodecyl sulfate-polyacrylamide gel electrophoresis (SDS-PAGE) analysis. The column was pretreated with lysis buffer (50 mM NaH_2_PO_4_, 300 mM NaCl, pH 8.0), and the proteins were eluted with elution buffer (50 mM NaH_2_PO_4_, 300 mM NaCl, 50 mM imidazole, pH 8.0). The protein samples were added to 10% glycerol (vol/vol) followed by storage at −80°C.

### Antibacterial activity test and growth curve assay

In this study, *E. coli* DH5α was used as an indicator strain for antibacterial activity testing. Antibacterial activity was determined using the Oxford Cup method as previously described (38). Strains were cultured at an initial OD_600_ of 0.7 in LB broth and transferred to the fermentation medium at a 1:33 dilution. Subsequently, 100 µL of cell-free supernatants was added to each hole, followed by culturing at 37°C for 12 h. All the treatments were prepared in triplicate.

Meanwhile, the quantitative analysis of polymyxin produced by *P. polymyxa* strains was performed using high-performance liquid chromatography-mass spectrometry (HPLC-MS) (Thermo Fisher, USA). The cell-free supernatants were precipitated four times with methanol at 4°C for 3 h and then centrifuged at 10,000 rpm, 4°C for 5 min. The supernatants were reserved and filtered through 0.22 µm membrane filters for HPLC-MS analysis. The HPLC system was set up as follows: Hola C18 column (2.1 mm × 100 mm, 1.9 µm); mobile phase A comprised water with 0.1% acetic acid, and organic phase B comprised acetonitrile with 0.1% acetic acid. The gradient elution procedure was as follows: 0 to 0.5 min, 90% A, 0.5 to 7 min, 0% A (decrement), 7 to 8.5 min, 0% A (isocratic), 8.6 min, 90% A, 8.6 to 10 min, 90% A (isocratic). The mass spectrometer was operated in a positive ion mode with a mass window ranging from 450 to 1,400 *m/z*. The areas of ion peaks at *m/z* 596.36 [M + 2H]^2+^ were obtained for quantitative polymyxin analysis (39).

Growth curves were determined by counting bacterial colony-forming units on LB agar plates every 2 h (2, 4, 6, 8, 10, 12, 14, and 16 h). Briefly, the SC2 strain was cultured in LB broth at an initial OD_600_ of 0.7 in the LB broth and transferred to the fermentation medium at a 1:33 dilution. Every 2 h, 100 µL of fermentation broth was serially diluted, and the dilutions (10^−4^, and 10^−5^) were spread onto LB solid medium using a glass spreading rod following incubation at 37°C for 24 h. Finally, bacterial colonies were counted.

### Electromagnetic mobility shift assay

The primers used for EMSA are listed in Table S1. Biotin-labeled DNA fragments were amplified from the gDNA of SC2 via PCR. EMSA was performed using a Chemiluminescent EMSA kit (Beyotime, China) following the manufacturer’s instructions. The purified proteins were incubated at increasing concentrations with biotin-labeled DNA fragments for 20 min at room temperature. The total reaction volume was 10 µL. Native-PAGE gel was used, and electrophoresis was performed at 70 V for 70 min using 0.5× Tris/Borate/EDTA buffer as the running buffer. The DNA and complexes were detected using an automatic molecular imaging system (FUSION SOLO S, Vilber GmbH, France).

### Construction of mutant strains

*abh* and *spo0A* in the SC2-M1 strain were deleted via homologous recombination. Briefly, the primer pairs abh-qF1/R1 and abh-qF3/R3 were used to amplify the upstream fragment 1 and downstream fragment 3 of *abh*, respectively. The primer pairs spo0A-qF1/R1 and spo0A-qF3/R3 were used to amplify the upstream fragment 1 and downstream fragment 3 of *spo0A*, respectively. The primer pairs abh-qF2/R2 and spo0A-qF2/R2 were used to amplify fragment 2 of the cat cassettes from plasmid pDG1661, respectively (40). Combined fragments 1–3 are generated using fusion PCR (41). Fragments 1–3 and the pRN5101 plasmid (Institute of Plant Protection, Chinese Academy of Agriculture Sciences, China) were digested (*Nhe*I/*Sal*I or *Nhe*I/*Bam*HI) and ligated overnight at 16°C and transformed into *E. coli* DH5α to obtain the deletion plasmids pRN5101-*abh* and pRN5101-*spo0A*. These plasmids were then transformed into *Trans* 110 cells to remove methylation and then electroporated into SC2-M1 cells. Double-crossover recombinants were used to screen knockout strains, and PCR was used for identification (19). Since *abrB3* in the SC2-M1 strain could not be deleted, the knockout and complementation strains for *abrB3* were not obtained.

### Construction of overexpression strains

First, the primer pairs abh-cF1/R1, abrB3-cF1/R1, and spo0A-cF1/R1 were used to amplify the promoter P*_04420_* from the gDNA of SC2 via PCR (42). The primer pairs abh-cF2/R2, abrB3-cF2/R2, and spo0A-cF2/R2 were used to PCR amplify the genes *abh*, *abrB3*, and *spo0A* from the gDNA of SC2, respectively. The primer pairs abh-cF3/R3, abrB3-cF3/R3, and spo0A-cF2/R2 were used to amplify cat cassettes from the plasmid pDG1661. Fusion fragments and pHY300PLK plasmid digested with *Bgl*II and *Sal*I were ligated using a one-step enzyme and transformed into *E. coli* DH5α and SC2-M1.

### Quantitative reverse transcription-PCR

Strains were cultured at an initial OD_600_ of 0.7 in LB broth, transferred to the fermentation medium at a 1:33 dilution, and then cultured at 37°C for 24 h before harvesting. Total RNA was extracted using the Total RNA Kit (Omega Bio-Tek, Norcross, GA, USA). Total RNA (800 ng) was reverse-transcribed into cDNA using the Evo M-MLV RT Kit (Accurate Biotechnology, Hunan, Co., Ltd., Changsha, China) according to the manufacturer’s instructions. qRT-PCR was performed using the SYBR Green Premix Pro Taq HS qPCR Kit (Accurate Biotechnology, Hunan, Co., Ltd., Changsha, China) according to the manufacturer’s instructions. The primers used for qRT-PCR in this study are listed in Table S1, and the concentration of primers used was 0.2 µM. Relative expression levels were determined using the 2^-ΔΔCt^ method with three technical replicates and three biological replicates (43).

### Data analysis

Promoter prediction was performed using Softberry (http://www.softberry.com/berry.phtml) (44). Sequence alignment was performed using DNAMAN. GraphPad Prism 9.0 software (La Jolla, CA, USA) was for generating plots. Primer Premier 5 was used for primer design. Statistical differences for different treatments were analyzed using the *t-test* of Microsoft Excel (Microsoft, Redmond, WA, USA). A value of *P* < 0.05 was considered significant.

## SUPPLEMENTAL MATERIAL

Supplemental figures spectrum.02293-23-s0001.docx Fig. S1 to S4.

Table S1 spectrum.02293-23-s0002.xlsx Bacterial strains, plasmids, and primers used in this study.

Table S2 spectrum.02293-23-s0003.xlsx Candidate binding protein bound to Ppmx identified by DNA pulldown assay.

ASM does not own the copyrights to Supplemental Material that may be linked to, or accessed through, an article. The authors have granted ASM a non-exclusive, world-wide license to publish the Supplemental Material files. Please contact the corresponding author directly for reuse.
