# Supplementary material for: Abh, AbrB3, and Spo0A play distinct regulatory roles during polymyxin synthesis in Paenibacillus polymyxa SC2
Source: Microbiol Spectr. 2023 Dec 6;12(1):e02293-23. doi: 10.1128/spectrum.02293-23 (PMC10782996; doi:10.1128/spectrum.02293-23)
Supplement: Supplemental figures — Fig. S1 to S4. [file spectrum.02293-23-s0001.docx]

Supplementary Material

Abh, AbrB3, and Spo0A play distinct regulatory roles during polymyxin synthesis in *Paenibacillus polymyxa* SC2

Yanru Cui, Dongying Zhao, Kai Liu, Xiangui Mei, Shanshan Sun, Binghai Du*, Yanqin Ding*

*** Correspondence:** Yanqin Ding: dyq@sdau.edu.cn. Binghai Du: du_binghai@163.com.


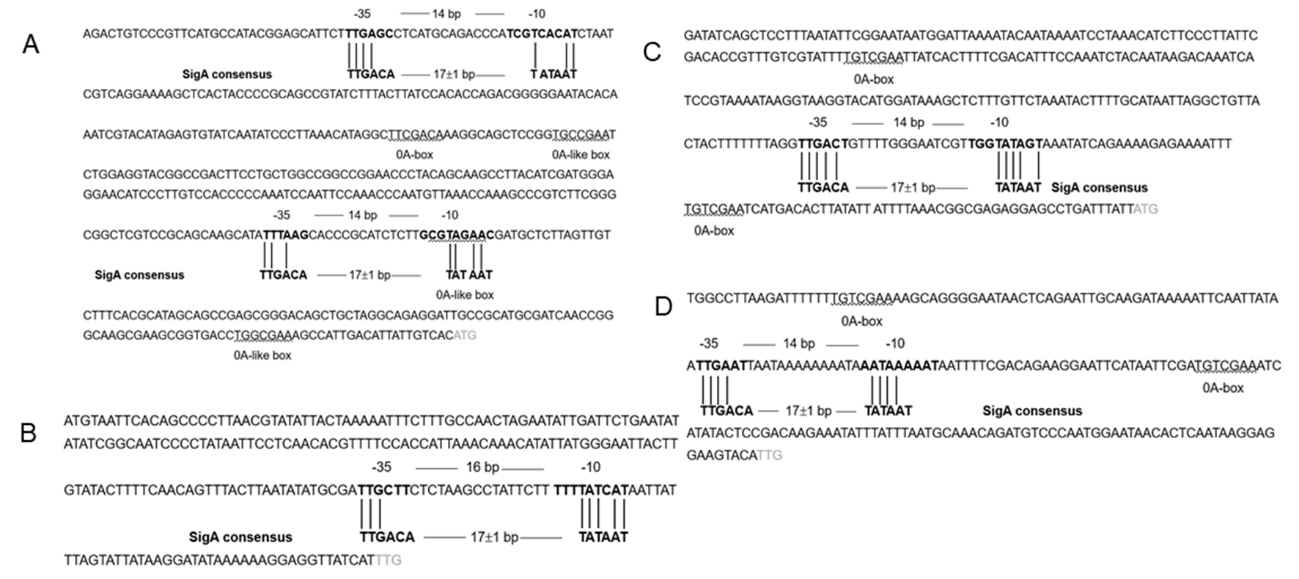


**FIG.S1.** Promoter analysis with Softberry. (A) The upstream 511 bp of *pmx* cluster, (B) the upstream 248 bp of *abh*, (C) the upstream 330 bp of *abrB3*, and (D) the upstream 216 bp of *spo0A* were analyzed with Softberry, respectively. Bold fonts represent -10 and -35 sequences and wavy lines represent 0A-box or 0A-like box. Grey fonts represent start codon. Comparison of upstream sequences of *pmx* cluster, *abh*, *abrB3*, and *spo0A* with consensus sequence of -10 and -35 regions of sigA-type promoters (Hoffmann et al., 2013).


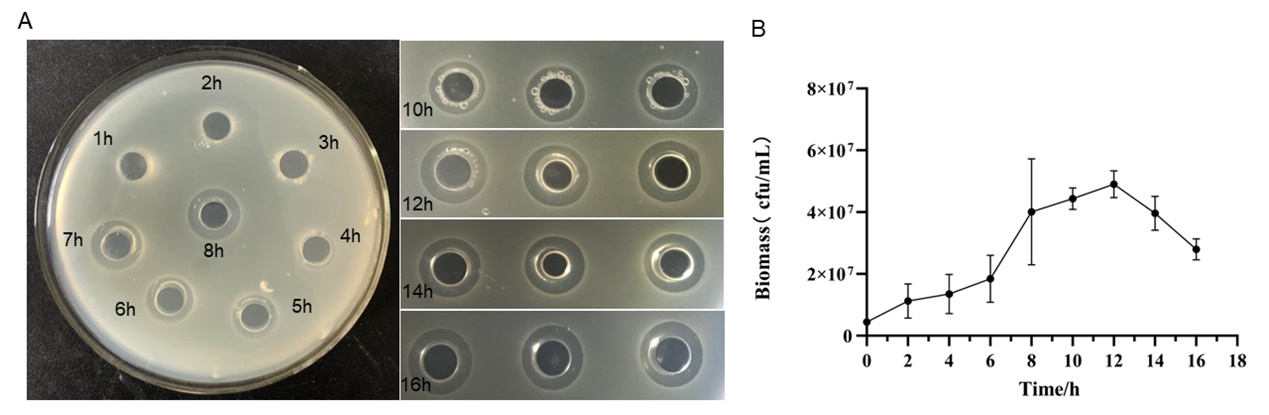


**FIG. S2.** Selection of the time for extracting SC2 total protein. (A) Antibacterial activity of SC2 fermentation broth at different times detected by using Oxford cup method. (B) The growth curve of *Paenibacillus polymyxa* SC2.


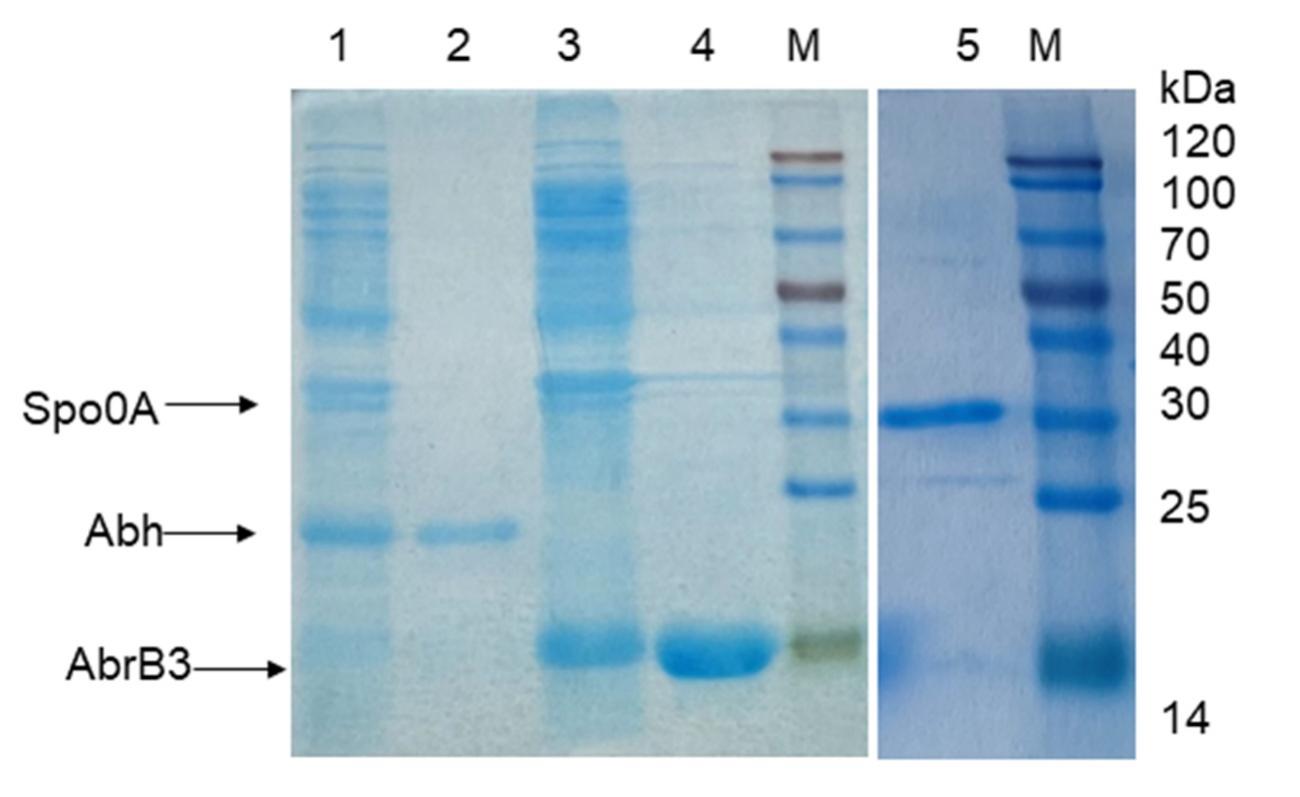


**FIG. S3.** SDS-PAGE analysis of protein Abh, AbrB, and Spo0A. M represents Marker, 1 and 3 represent total protein; 2 represents purified proteins His6-Abh; 4 represents purified proteins His6-AbrB3, and 5 represents purified proteins His6-Spo0A.


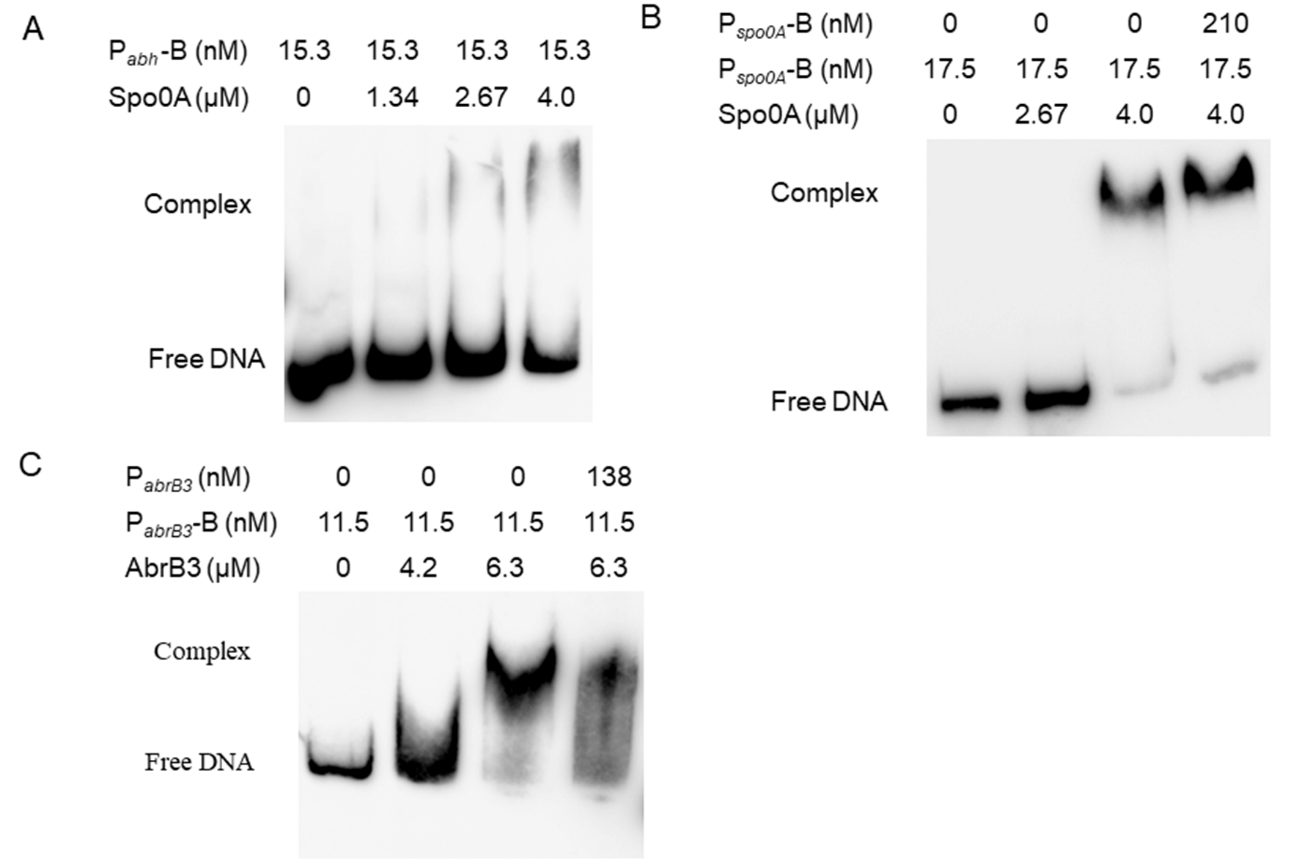


**FIG. S4.** Abh, Spo0A and AbrB3 binding to P*_abh_*, P*_spo0A_*, and P*_abrB3_* dected by EMSA. (A) The increasing concentrations of purified Spo0A (0, 1.34, 2.67, and 4.0 μM) binds to P*_abh_* (15.3 nM) were analyzed by EMSA. (B) The increasing concentrations of purified Spo0A (0, 2.67, and 4.0 μM) binding to P*_spo0A_* (17.5 nM) were analyzed by EMSA. (C) The increasing concentrations of purified AbrB3 (0, 4.2, and 6.3μM) binding to P*_abrB3_* (11.5 nM) were analyzed by EMSA.
